# Human peripheral blood mononuclear cells (PBMCs) from smokers release higher levels of IL-1-like cytokines after exposure to combustion-generated ultrafine particles

**DOI:** 10.1038/srep43016

**Published:** 2017-02-22

**Authors:** Gianluigi De Falco, Michela Terlizzi, Mariano Sirignano, Mario Commodo, Andrea D’Anna, Rita P. Aquino, Aldo Pinto, Rosalinda Sorrentino

**Affiliations:** 1 Dipartimento di Ingegneria Chimica, dei Materiali e della Produzione Industriale, Università degli Studi di Napoli Federico II, Piazzale V. Tecchio, 80, Napoli, Italy; 2 Istituto di Ricerche sulla Combustione – National Research Center (CNR), Piazzale V. Tecchio, 80, Napoli, Italy; 3 Department of Pharmacy (DIFARMA), University of Salerno; ImmunePharma s.r.l., Via Giovanni Paolo II 132, Salerno, Italy

## Abstract

Ultrafine particles (UFP) generated by combustion processes are often associated with adverse health effects. However, little is known about the inflammatory processes generated by UFP that may underlie their toxicological activity. Murine macrophages (J774.1 cells) and human peripheral blood mononuclear cells (PBMCs) were used to evaluate the molecular mechanism underlying the pro-inflammatory activity of UFP. The addition of soot particles to J774.1 cells induced a concentration-dependent release of IL-1α, IL-1β and IL-33 This effect was not associated with cell death and, in contrast to literature, was pronounced at very low concentrations (5–100 pg/ml). Similarly, UFP induced the release of IL-1α, IL-18 and IL-33 by PBMCs. However, this effect was solely observed in PBMCs obtained from smokers, as the PBMCs from non-smokers instead released higher levels of IL-10. The release of these cytokines after UFP exposure was caspase-1- and NLRP3 inflammasome-dependent in PBMCs from healthy smokers, whereas IL-1α release was calpain-dependent. These results show that UFP at very low concentrations are able to give rise to an inflammatory process that is responsible for IL-1α, IL-18 and IL-33 release, which is pronounced in PBMCs from smokers, confirming that these individuals are especially susceptible to inflammatory-based airway diseases once exposed to air pollution.

Epidemiological studies have widely demonstrated a direct link between air pollution and respiratory diseases. Diesel exhaust particles represent one of the major environmental insults responsible for adverse effects on the respiratory tract^1^,^2^,^3^. Combustion particles emitted by diesel engines consist of fine particles often referred to as soot^4^; in particular, sub-100 nm particles (ultrafine particles, UFP) are the most threatening, as they can localize into the low tract of the respiratory tree, leading to pulmonary diseases^3^. Several studies demonstrated that exposure to soot particles has remarkable effects on the immune system^5^,^6^,^7^,^8^. However, most of the studies are focused on allergic diseases^9^,^10^. In this regard, it was demonstrated that exposure to soot particles causes changes in lymphocyte homeostasis and immune responses in that it promotes autophagy in T cells with a Th2-like phenotype^11^. In recent years, dysfunctional autophagy has been linked to inflammatory pathways that promote oxidative stress and DNA damage and mutations, phenomena that can lead to cancer development^12^. Nevertheless, the exact mechanism underlying soot particle-induced immune cell dysfunction with ensuing inflammation, which can ultimately lead to toxicological effects on human health, is yet unknown. It was described that UFP are able to induce both epithelial and macrophagic cells to release reactive oxygen species (ROS), which are responsible for the induction of cell death via apoptosis and/or necrosis^13^,^14^,^15^. In this scenario, the role of mitochondria is pivotal in that alterations in the membrane depolarization-hyperpolarization equilibrium can promote the release of mitochondrial ROS (mtROS), which have recently been described as potential inducers of inflammatory pathways^16^. Shimada *et al*.^17^ demonstrated that the release of mtDNA together with mtROS can induce the activation of the inflammasome complex. The inflammasome is a multiprotein complex that is generated from the assembly of Nucleotide-binding oligomerization domain (Nod)-like receptors (NLRs) or HIN200 family receptors, such as absent in melanoma 2 (AIM2), which are able to bind to the adaptor apoptosis-associated speck-like protein containing a carboxy terminal CARD (ASC), which induces the autocleavage of caspase-1; this promotes the release of IL-1-like cytokines in their active form^18^. The activation of the inflammasome leads to the release of IL-1-like cytokines, which are responsible for early inflammation and can result in long-term, chronic inflammation^18^. In support of this, IL-1-like cytokines are highly detected in the serum of cancer patients. Here, UFP produced under controlled conditions in laboratory were characterized in terms of their ability to modify inflammatory cytokine production in human peripheral blood mononuclear cells (PBMCs). In contrast to the literature data, where the pro-inflammatory activity of soot particles is associated with concentrations on the order of μg/ml, combustion-generated soot particles were tested for the ability to induce IL-1-dependent inflammation at very low concentrations (pg/ml). Here, we found that combustion particle-treated PBMCs obtained from smoking, but not non-smoking, healthy volunteers were able to release IL-1α, IL-18 and IL-33 in a caspase-1-dependent manner. In contrast, PBMCs from non-smokers showed higher release of the immunosuppressive IL-10, suggesting host defence against the activity of IL-1-like cytokines. In contrast, although PBMCs from smokers still released IL-10 after the addition of soot particles, this effect was less pronounced than that in non-smokers, implying that both smoking and air pollution can prompt pulmonary inflammation in an IL-1-like manner.

Our data provide new perspectives for investigation into the role of inhaled combustion particles that, together with other pulmonary insults, can lead to the inflammation that may underlie lung pathologies such as asthma, lung fibrosis and cancer.

## Results

### Ultrafine soot particles induced a concentration-dependent release of IL-1-like cytokines in murine macrophages

**Figure 1:**
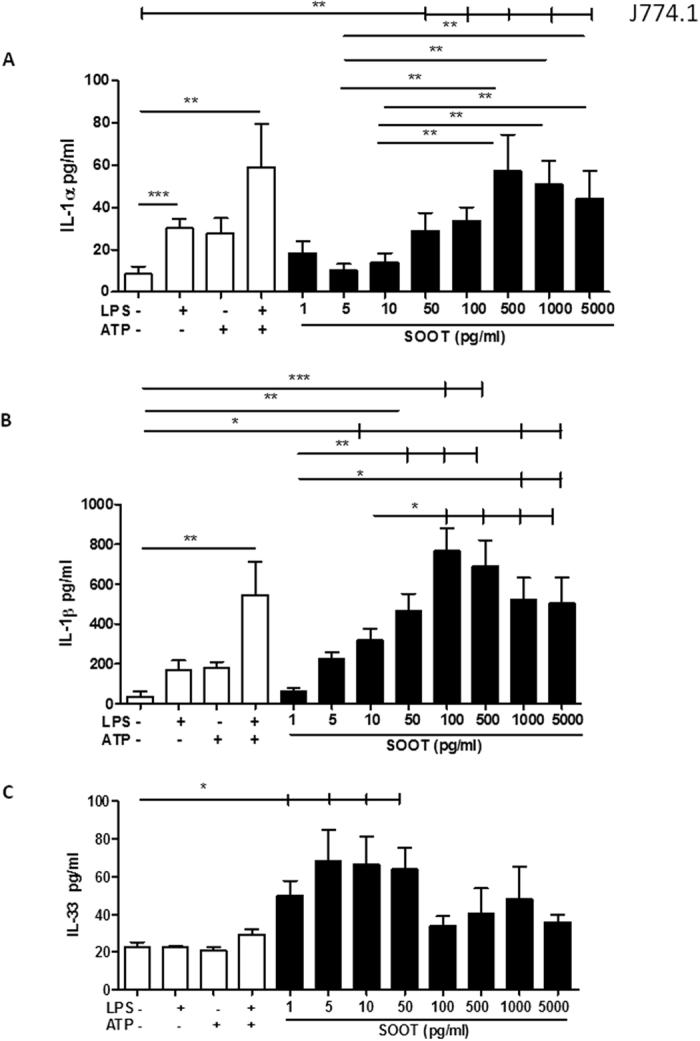
The administration of soot particles for 5 hours induced the release of IL-1-like cytokines by murine macrophages. J774.1 cells (a murine macrophage cell line) were treated with soot particles in a concentration-dependent manner (1 pg/ml–5 ng/ml) for 5 hours. LPS (0.1 μg/ml) and/or ATP (0.5 mM) was used as a positive control. The addition of soot particles to macrophages induced the release of IL-1β (**A**), IL-1α (**B**) and IL-33 (**C**). Data are presented as the means ± SEM (n = 12). Statistically significant differences are denoted by *, ** and ***, indicating p < 0.05, p < 0.01 and p < 0.001, respectively, as determined by one-way ANOVA followed by Bonferroni’s multiple comparison post-test.

Combustion-generated UFP have been widely demonstrated to be one of the main insults on human health^3^. To study the under-investigated potential pro-inflammatory activity of soot particles, we preliminarily considered an early step in the induction of inflammation, the release of IL-1-like cytokines, such as IL-1α, IL-1β, IL-18 and IL-33, in the J774.1 macrophagic/monocytic cell line. J774.1 cells were treated with the produced UFP in a concentration-dependent manner starting at 1 pg/ml and increasing up to 5 ng/ml. As shown in Fig. 1, soot particles were able to statistically increase the release of IL-1α (Fig. 1A), IL-1β (Fig. 1B) and IL-33 (Fig. 1C), but not IL-18 (data not shown), after 5 hours of treatment. However, it is worth noting that this effect was observed at concentrations much lower than what has been reported in the literature, which has described the pro-inflammatory and cytotoxic activities of diesel exhaust particles/air pollution-derived particles at concentrations of μg/ml^7^,^19^,^20^,^21^. Importantly, the bell-shaped release of IL-1α (Fig. 1A), IL-1β (Fig. 1B) and IL-33 (Fig. 1C) showed that the sub-maximal effect was reached at concentrations of 5, 10, 50 and 100 pg/ml rather than higher concentrations (500, 1000 and 5000 pg/ml). Notably, UFP treatment of cells stimulated the release of the cytokines at similar or higher levels than those observed for positive controls (LPS ± ATP).

To understand the effect of UFP on other pro-inflammatory cytokines, the release of IL-6 and TNF-α was evaluated. As shown in Fig. 1 of the Supplementary Material, soot particles were not able to induce the release of IL-6 (Supplementary Figure 1A) and TNF-α (Supplementary Figure 1B) at our working concentrations. However, particles were able to induce IL-10 release (data not shown), implying that the phenotype of macrophages tended towards an M2 polarization.

A 3-(4,5-dimethylthiazol-2-yl)-2,5-diphenyltetrazolium bromide (MTT) test showed that J774.1 macrophagic/monocytic cells were viable after the addition of soot particles at both 5 hours (Supplementary Figure 2A) and 24 hours (Supplementary Figure 2B). To rule out any interference of soot particles to absorb at 550 nm, we also performed the MTT assay in a cell-free medium after the addition of the sole soot particles at the concentration of 1 pg/ml−5 ng/ml. Soot particles did not alter the absorbance of the MTT assay compared to the sole medium without particles (Supplementary 2C). To further confirm this observation, cells were treated with necrostatin, a well-known inhibitor of receptor-interacting protein (RIP) kinase involved in necrosis/cell death^22^. The addition of necrostatin did not change the levels of IL-1-like cytokines (data not shown), implying that soot particles do not induce necrosis. Moreover, soot particles were not able to induce the cleavage of caspase-3 (data not shown), a well-known enzyme involved in the apoptotic cascade.

These data imply that soot particles induce early inflammation in J774.1 cells via the release of IL-1-like cytokines without affecting cell viability.

### Ultrafine soot particles induced a concentration-dependent release of IL-1-like cytokines from PBMCs from smokers

To verify if the observed effects on J774.1 cells were also valid for human primary cells, the effect of combustion-generated soot particles was evaluated in PBMCs isolated from healthy volunteers distinguished as non-smokers and smokers.

**Figure 2:**
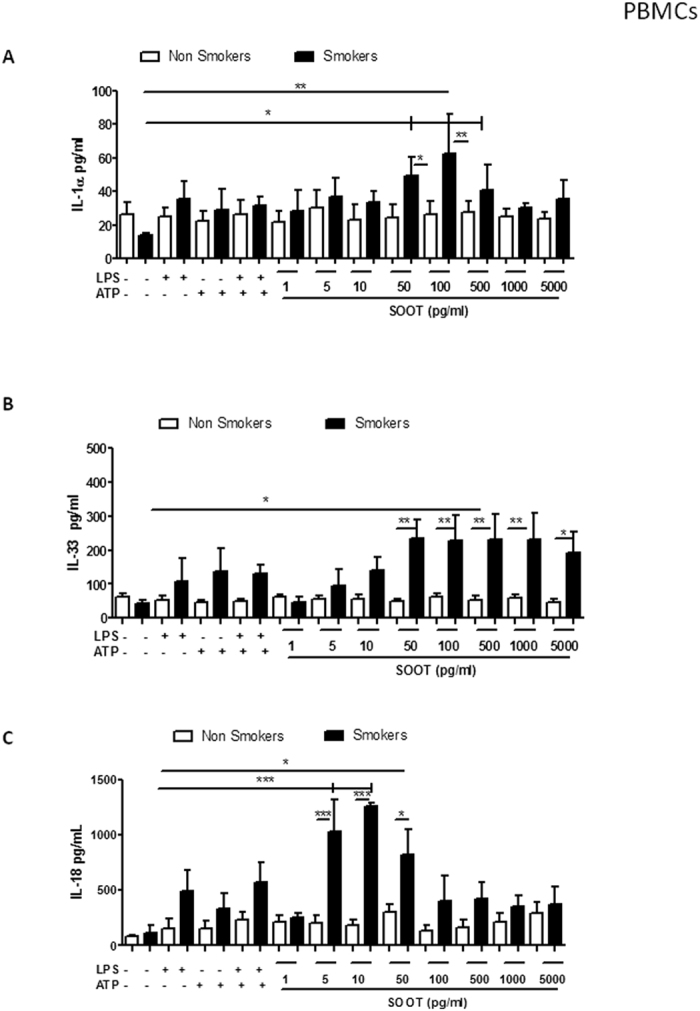
PBMCs obtained by healthy smokers were more susceptible to soot particle-induced IL-1-like cytokine release. PBMCs were isolated from the blood of healthy non-smoking and smoking volunteers. The addition of soot particles (1 pg/ml–5 ng/ml) for 5 hours to PBMCs isolated from non-smokers did not induce the release of IL-1α (**A**) or IL-33 (**C**). However, soot particles slightly increased the release of IL-18 (50 pg/ml) (**E**). In sharp contrast, the addition of soot particles to PBMCs obtained from smokers significantly increased the release of IL-1α (**B**), IL-33 (**D**) and IL-18 (**F**). Data are presented as the means ± SEM (n = 5). Statistically significant differences are denoted by *, ** and ***, indicating p < 0.05, p < 0.01, p < 0.001 and p < 0.001, respectively, as determined by one-way ANOVA followed by Bonferroni’s multiple comparison post-test.

**Figure 3:**
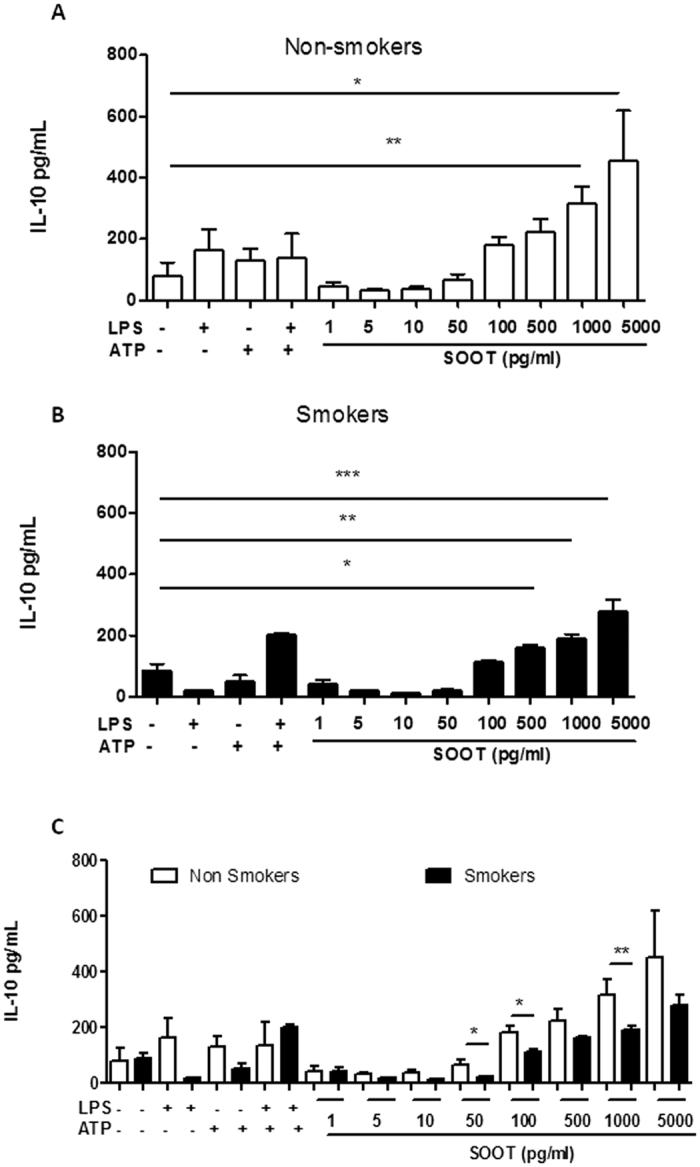
The addition of soot particles induced PBMCs to release IL-10. PBMCs were isolated from the blood of healthy non-smoking and smoking volunteers. The administration of soot particles (1 pg/ml–5 ng/ml) for 5 hours to PBMCs isolated from non-smokers significantly increased the release of IL-10 (**A**). In the same way, but to a lower extent, PBMCs isolated from smokers released IL-10 after soot particle addition (**B**). (**C**) Comparison of the levels of IL-10 between non-smokers (open white bars) and smokers (dotted bars). Data are presented as the means ± SEM (n = 5). Statistically significant differences are denoted by *, ** and ***, indicating p < 0.05, p < 0.01 and p < 0.001, respectively, as determined by one-way ANOVA followed by Bonferroni’s multiple comparison post-test.

Interestingly, the addition of UFP to PBMCs obtained from healthy non-smokers did not induce the release of IL-1α (Fig. 2A, white bars), IL-33 (Fig. 2B, white bars) or IL-18 (Fig. 2C, white bars). In contrast, the addition of UFP (1 pg/ml–5 ng/ml) to PBMCs from healthy smokers significantly increased the release of all the cytokines (IL-1α, IL-33 and IL-18; Fig. 2A,B and C, respectively, dotted bars). Again, the release of these cytokines in response to treatment with soot particles was similar to or higher than the cytokine response of the positive control (smokers: LPS ± ATP, dotted bars). To understand the effect of soot particles on PBMCs from non-smokers, we analysed the release of an immunosuppressive cytokine, IL-10^23^, the level of which was significantly increased (Fig. 3A). Similarly, PBMCs obtained from smokers released IL-10 (Fig. 3B). However, it is important to note that the level of this cytokine was higher for PBMCs obtained from non-smokers (Fig. 3C, white bars) than PBMCs from smokers (Fig. 3C, dotted bars) after treatment with soot particles.

The combined results imply that smokers exposed to UFP at very low concentrations are more susceptible to IL-1-dependent inflammation than non-smokers.

### The release of IL-1-like cytokines after treatment of smoker-derived PBMCs with ultrafine soot particles was NLRP3/caspase-1-dependent

IL-1-like cytokine release is strictly dependent on the multimeric inflammasome complex^18^,^22^. To understand the molecular mechanism underlying the release of these cytokines after soot particle exposure, we tested the effect of well-known pharmacological inhibitors.

**Figure 4:**
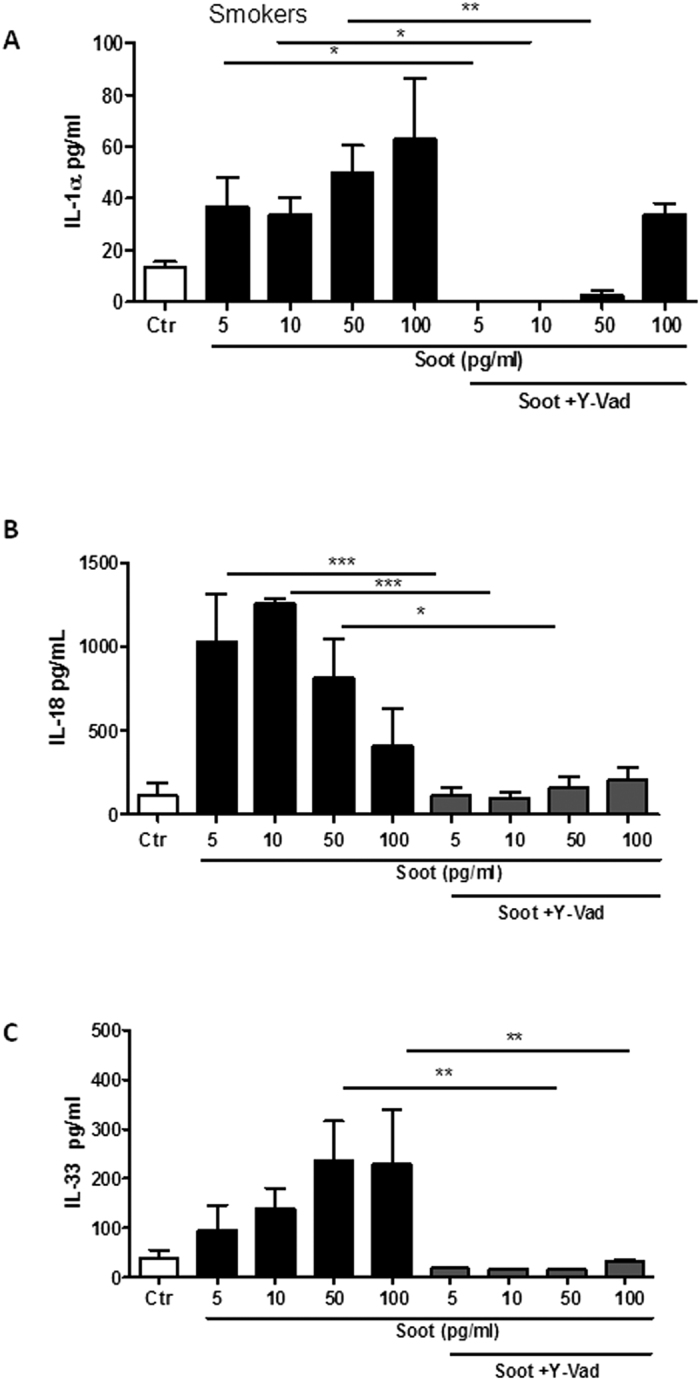
The release of IL-1-like cytokines by soot particle-treated PBMCs obtained from healthy smokers is caspase-1-dependent. PBMCs obtained from smokers were treated with soot particles (1 pg/ml–5 ng/ml) in the presence or absence of Ac-YVad (Y-Vad: 1 μg/ml), a caspase-1 inhibitor. The inhibition of caspase-1 with Y-Vad significantly reduced the release of IL-1α (**A**), IL-18 (**B**) and IL-33 (**C**) from PBMCs treated for 5 hours. Data are presented as the means ± SEM (n = 5). Statistically significant differences are denoted by *, ** and ***, indicating p < 0.05, p < 0.01, and p < 0.001, respectively, as determined by one-way ANOVA followed by Bonferroni’s multiple comparison post-test.

**Figure 5:**
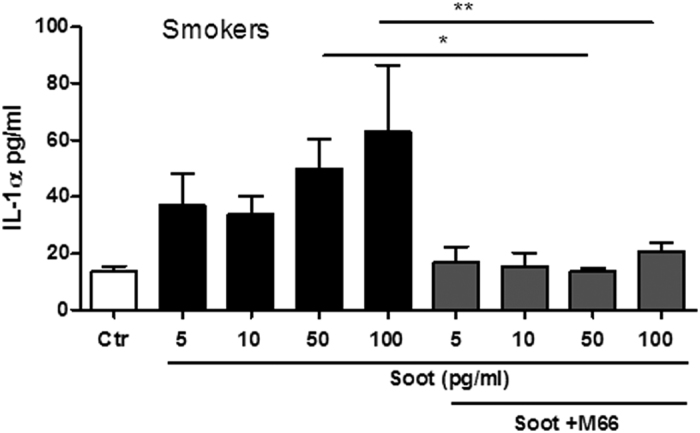
NLRP3 is involved in the release of IL-1-like cytokines by soot particle-treated PBMCs obtained from healthy smokers. PBMCs obtained from smokers were treated with soot particles (1 pg/ml–5 ng/ml) in the presence or absence of glybenclamide (Gly: 1 μM), an NLRP3 inhibitor. The inhibition of NLRP3 with Gly significantly reduced the release of IL-1α (**A**), IL-18 (**B**) and IL-33 (**C**) from PBMCs treated for 5 hours. Data are presented as the means ± SEM (n = 5). Statistically significant differences are denoted by * and ***, indicating p < 0.05 and p < 0.001, respectively, as determined by one-way ANOVA followed by Bonferroni’s multiple comparison post-test.

Cells were co-treated with soot particles and Y-Vad, a caspase-1 inhibitor. The administration of Y-Vad significantly decreased the release of all cytokines (IL-1α, IL-18 and IL-33 (Fig. 4A,B and C, respectively). However, higher concentrations of soot particles (100 pg/ml) still induced the release of IL-1α (Fig. 4A) after the addition of Y-Vad, implying that another mechanism was involved. Because IL-1α release is also dependent on the activation of calpain I/II^22^, we analysed whether this pathway was involved. Cells from smokers were treated with M66, a calpain I/II inhibitor^22^. The addition of M66 completely inhibited the release of IL-1α (Fig. 5) after treatment with soot particles, even at higher concentrations (50–100 pg/ml).

**Figure 6:**
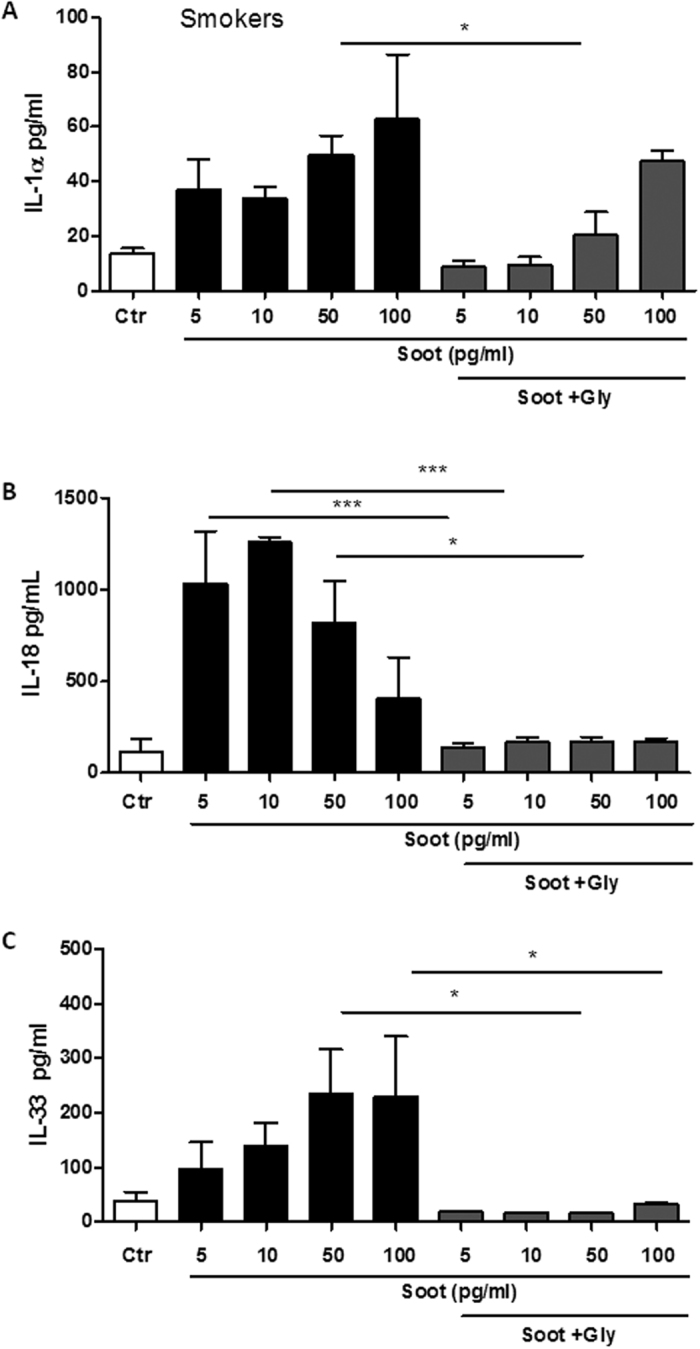
The release of IL-1-α from soot particle-treated PBMCs obtained from healthy smokers is calpain-dependent. PBMCs obtained from smokers were treated with soot particles (1 pg/ml–5 ng/ml) in the presence or absence of M6690 (M66: 10 μM), a calpain I/II inhibitor. The inhibition of calpain I/II significantly reduced the release of IL-1α from PBMCs treated for 5 hours. Data are presented as the means ± SEM (n = 5). Statistically significant differences are denoted by * and **, indicating p < 0.05 and p < 0.01, respectively, as determined by one-way ANOVA followed by Bonferroni’s multiple comparison post-test.

Because the inflammasome complex comprises an intracellular receptor that assembles with ASC and caspase-1, the involvement of the NLRP3 inflammasome was examined. PBMCs from smokers were treated with soot particles in the presence of glybenclamide, a well-known inhibitor of the NLRP3 inflammasome^22^. The inhibition of NLRP3 significantly reduced the release of the cytokines IL-1α, IL-18 and IL-33, as reported in Fig. 6A–C. Similarly, IL-1α release was not completely reduced at the higher concentration of 100 pg/ml (Fig. 6A), confirming previous data (Fig. 4A) suggesting that other molecular mechanisms may be involved in IL-1α release (Fig. 5).

These data support the hypothesis that the inflammasome plays a critical role in the inflammatory effect of soot particles on PBMCs from smokers.

## Discussion

In this study, we found that human PBMCs derived from healthy smokers are more susceptible to ultrafine soot particle-induced IL-1-dependent inflammation. We found that treating PBMCs from smokers with soot particles induced the activation of the NLRP3 inflammasome, leading to caspase-1 activation and the ensuing release of IL-1α, IL-18 and IL-33, which are responsible for the pro-inflammatory activity of soot particles. The effect was observed at concentrations (50, 100 and 500 pg/ml) of soot particles much lower than those already reported in the literature (μg/ml)^7^,^19^,^20^,^21^. Instead, higher concentrations of soot particles led to the release of immunosuppressive IL-10 from PBMCs from both smokers and non-smokers, although IL-10 was higher from non-smokers. Moreover, in sharp contrast with previous studies^24^,^25^, the administration of soot particles did not lead to cell death.

IL-1α is an alarmin that under physiological conditions ‘instructs’ the adaptive immune system to trigger sterile inflammation^18^. Likewise, IL-1β and IL-18, according to the microenvironment, can promote T cell survival and the polarization of Th1, Th2 and Th17 cells and mediate leukocyte migration^26^. The results of this study showed that soot particles were able to induce the release of these cytokines at concentrations ranging from 5 pg/ml up to 100 pg/ml. In support of this, Bengalli *et al*.^27^ showed that treating a human monocyte cell line, THP-1 cells, with fine (10 nm) particulate matter caused the release of IL-1β in an NLRP3 inflammasome-dependent manner due to the recognition of particles by Toll-like receptors (TLRs) 2 and 4, which are first activated and stimulate the induction of a second signal, NLRP3 activation, that promotes the assembly of the inflammasome complex. Similarly, we found that soot particles can lead to IL-1α, IL-18 and IL-33 release via the activation of NLRP3 and caspase-1. However, the effect was observed only for PBMCs from smokers, while no effect could be observed for PBMCs from healthy non-smokers. Moreover, this effect was observed at concentrations much lower than what has been reported in the literature.

In addition, soot particles were able to release IL-33 from both macrophagic cells (J774.1) and PBMCs obtained from smokers. IL-33 is an alarmin of the IL-1 family that is crucial for the innate immune response, as it regulates immune cell infiltration and activation^18^.

The release of IL-1-like cytokines promotes the activation of NF-κB^28^, which is at the basis of inflammatory pathways. In support of this, several authors demonstrated that soot particles can lead to NF-κB activation in both macrophage/monocyte cell lines and a human epithelial cell line (A549)^29^,^30^.

According to the sterile inflammation theory, sterile, noninfectious insults, such as reactive oxygen species (ROS), oxidized and/or methylated DNA, high-mobility group box 1 (HMGB1), heat shock proteins, and ATP, all of which are generally identified as damage-associated molecular patterns (DAMPs), can induce chronic inflammation^18^. All these stimuli can behave as tumour promoters, as their underlying activity is the induction of chronic inflammation that, rather than providing a protective response to the loss of tissue homeostasis, can aberrantly facilitate tumour development. These insults, both endogenous and exogenous, are sensed by the inflammasome^31^. Our results demonstrate that UFP at very low concentrations can lead to NLRP3 inflammasome activation, which is responsible for the release of IL-1-like cytokines. However, it is very interesting that this phenomenon was observed only in PBMCs from smokers and not those from non-smokers, which instead showed immunosuppressive behaviour. Therefore, it is tempting to speculate that smokers, a high-risk population, exposed to ultrafine soot particles may be more susceptible to lung cancer than non-smokers. The inhalation of particulate matter together with smoking derivatives can lead to cell transformation, which is favoured by a pro-inflammatory scenario as in the case of IL-1-like cytokine release. In particular, because we observed that the release of pro-inflammatory cytokines occurs at low concentrations of UFP, it is likely that, *in vivo*, the presence of low amounts of soot particles in the respiratory tract together with smoking can induce adverse toxicological effects, such as cell transformation, in contrast to what has so far been found using high amounts (on the order of μg/ml) of soot particles. However, cell transformation occurs in epithelial cells, whereas our study was conducted on PBMCs, which are leukocytes. However, it is well-known that the immune system plays a pivotal role in tumour immune escape in that the immune cells are tolerogenic and therefore incapable of recognizing transformed cells as non-self^32^. In this context, immunosuppression overtakes the immunostimulatory activity of innate immune cells such as dendritic cells and macrophages, which in their tolerant phenotype can facilitate the immunosuppressive arm of the adaptive immune system^22^. In support of this, we found that higher concentrations of soot particles (500 pg/ml–5 ng/ml) were able to induce the release of IL-10 from macrophages and PBMCs. IL-10-producing macrophages are reported to be M2 polarized cells that, in the context of tumours, can facilitate tumour progression, confirming the hypothesis about the role of soot particle-induced IL-1-like cytokines in the tumour microenvironment^33^.

Another issue to consider is the absence of cell death after treating both macrophages and PBMCs with soot particles. We found that neither J774.1 cells nor PBMCs underwent cell death after 5 and 24 hour of treatment with soot particles. These data are in contrast with literature data^24^,^25^. One explanation is that all the studies conducted so far have been performed with higher concentrations (μg/ml) of soot particles than our experimental conditions (1 pg/ml up to 5 ng/ml). In support of this, Pierdominici *et al*.^11^ demonstrated that T lymphocytes were not induced to undergo apoptosis or necrosis after soot particle treatment; rather, they found that the autophagy machinery was activated due to alterations in the mitochondrial membrane potential. Indeed, ROS are highly produced after the administration of soot particles. Several studies have reported that high concentrations of soot particles lead to the release of ROS, with subsequent cell death^13^,^14^,^15^. Similar to the findings of Pierdominici *et al*.^11^, we did not observe cell death after soot particle addition. The production of ROS by mitochondria is one of the signals that is able to induce NLRP3 inflammasome activation^34^. Therefore, although we still do not know whether soot particles activate the inflammasome in a direct or indirect manner, we can confirm that the activation of this complex leads to the release of not only IL-1β, as already reported, but also other alarmins, such as IL-1α, IL-18 and IL-33. Moreover, it is worth noting that IL-1α was induced not only after the activation of the inflammasome but also by the induction of the calpain machinery. Gross *et al*.^35^ demonstrated that the activation of the NLRP3, NLRC4 and absent in melanoma 2 (AIM2) inflammasomes can lead to the release of IL-1α, but not in a direct manner. In this scenario, the authors showed that IL-1α was not universally inflammasome dependent but rather was dependent on calcium-dependent calpain protease activity. Supporting this paper is the fact that IL-1α is not a substrate of caspase-1, although some danger molecules can regulate the inflammasome-dependent processing and release of IL-1α in mouse bone-marrow-derived macrophages. In this context, we found that calpain is involved in soot particle-induced IL-1α release by PBMCs obtained from healthy smokers, in contrast to the results for non-smokers. However, based on our data, it is most likely that the addition of IL-1α was primarily dependent on the activation of calpain I/II, which is calcium-dependent. It is worthy to note that UFP were reported to induce intracellular calcium transients^36^. Moreover, in our previous study, we found that plasmacytoid dendritic cells (pDCs) derived from the cancerous lesions of patients with lung cancer were able to release IL-1α, rendering pDCs tolerogenic in that they highly populated tumour lesions, thus implying their role in tumour proliferation^22^. Similarly, PBMCs from smokers, a population well known to be at high risk of lung cancer, released higher levels of IL-1α and IL-10 than PBMCs from non-smokers, implying that the release of both types of cytokines may favour cell transformation and thus carcinogenesis. However, the levels of IL-1α were not as high as that for the other cytokines (IL-33 and IL-18). This issue could be due to the limits of the ELISA kit, or it could imply that even low concentrations have biological impacts. In support of this, Cohen *et al*. found that UV-irradiated cells released approximately 200 pg/ml of IL-1α, which in this paper is considered to be an alarmin that signals genotoxic stress^37^. Similarly, in our previous study^22^, we found that approximately 150–200 pg/ml of IL-1α were released from cancerous cells after stimulation. In contrast, healthy cells had a basal release of IL-1α at approximately 20 pg/ml, while the release from lung cancer cells was higher (~34 pg/ml), suggesting that even low (detectable) levels can be involved in lung pathogenesis. Based on this result, we believe that the statically significant increase in IL-1α from PBMCs post soot particle treatment can interfere with host immune responses. It is to note though, that treatment of cells with recombinant IL-1α needed higher concentrations (approximately μg/ml) of this cytokine to observe a biological effect in *in vitro* studies.

In conclusion, our study highlights the molecular mechanism by which very small nanoparticles induce the release of more IL-1α, IL-18 and IL-33 in smoking individuals than non-smokers, who instead showed higher release of the immunosuppressive cytokine IL-10, implying host defence against the pro-inflammatory activity of IL-1-like cytokines. In contrast, although PBMCs from smokers released IL-10 after the addition of soot particles at high concentrations (500 pg/ml–5 ng/ml), the levels of IL-10 were lower than those in non-smokers, implying that both smoking and air pollution can induce pulmonary inflammation in an IL-1-like manner.

The findings of the present study demonstrate the molecular mechanism that underlies the pronounced susceptibility of smokers to inflammatory-based airway diseases once exposed to air pollution. Human PBMCs derived from healthy smokers are more susceptible to ultrafine soot particle-induced IL-1-dependent inflammation via activation of the NLRP3 inflammasome, which leads to caspase-1 activation and the ensuing release of IL-1α, IL-18 and IL-33.

Our data provide new perspectives for the investigation of the role of inhaled combustion ultrafine particles that together with other pulmonary insults can lead to inflammation that may underlie allergic diseases, lung fibrosis and lung cancer. Taken together, these data provide new insight into pulmonary inflammation that can be induced by very small nanoparticles after exposure to air pollution.

## Materials and Methods

### Human Samples

We used blood from healthy volunteers (smokers and non-smokers) recruited at the “Monaldi-Azienda Ospedaliera (AORN)-Ospedale dei Colli” Hospital in Naples, Italy, after their approval according to the Review Board of the hospital and the patients’ informed consent. In addition, all experimental protocols were, as stated above, approved by the Review Board of “Monaldi-AORN-Ospedale dei Colli” Hospital (approval number 1254/2014). In addition all experimental protocols were performed in accordance with the guidelines and regulations provided and accepted by the Review Board of the “Monaldi-AORN-Ospedale dei Colli” Hospital. The healthy volunteers were 50 ± 10 years of age. All subjects had no history of allergic diseases or chronic respiratory conditions. Blood was collected and used within 24 hours.

### Isolation of human PBMCs

Mononuclear cells were isolated according to Ficoll’s protocol. Briefly, blood (5 ml) was mixed with cell medium (5 ml) supplemented with sole antibiotics and Ficoll medium (Life Sciences, Italy). Then, it was centrifuged at 1125 g for 20 minutes. The PBMC layer was collected, diluted with cell medium and centrifuged at 753 g to remove the remaining Ficoll solution. Platelets were separated from the PBMCs by centrifugation at 149 g for 10 minutes. PBMCs were collected in cell medium and plated.

### Cell culture

Macrophagic cells, J774.1 cells, and PBMCs were cultured in Roswell Park Memorial Institute (RPMI) 1640 (Cambrex Biosciences, Microtech, Naples, Italy) supplemented with 10% heat-inactivated foetal bovine serum (FBS), 100 units/ml penicillin, 100 units/ml streptomycin and 2 mM L-glutamine (Cambrex Biosciences, Microtech, Naples, Italy). Cells were seeded and treated with LPS (0.1 μg/ml, Alexis, Vincibiochem, Italy), ATP (0.5 mM, Sigma Aldrich, Italy), soot particles (1 pg/ml–5 ng/ml), Ac-YVad (Y-Vad: 1 μg/ml), or M6690 (M66: 10 μM). Cells were treated for 5 or 24 hours, as reported in the Results section.

### Soot samples

Soot particles were collected at the exhaust of an atmospheric pressure acetylene/air premixed flame operated in fuel-rich conditions (equivalence ratios greater than 3). The flame was ignited on a McKenna burner, and it was stabilized on the burner by a metal plate located approximately 3 cm from the burner outlet. The soot particles deposited on the metal plate after some hours of flame operation were removed by mechanical ablation and collected as a powder. The obtained sample was washed with dichloromethane to remove the organic fraction condensed on the particles, dried and used for spectroscopic, elemental and morphologic analyses. Ultraviolet-visible (UV-vis) and infrared spectroscopy and Raman spectroscopy were used for structural analysis of the carbon-network constituting the soot particles^38^. Soot particles appeared as a network of aromatic structures with few peripheral H-atoms. The size of the aromatic network was relatively small, as the dimension of the polyaromatic subunits retrieved from Raman spectroscopy was smaller than 2 nm. Elemental analysis confirmed the low presence of H atoms in the soot particles, as the amount of C was approximately 96%, in mass, of the total material, and H was approximately 2.5–2.8%, with the rest being trace compounds. Atomic force microscopy of the material deposited directly from the flame on mica disks and before any further sample treatment showed that the soot particles were made up of spherical particles with sizes on the order of 20–30 nm and larger, chain-like, soot agglomerates with sizes up to a few hundred nanometers^39^,^40^.

### Sample preparation

Soot particles were dispersed in bidistilled water to obtain a suspension with a concentration of 5 ppm (5 μg/ml). This constituted the pristine sample from which different concentrations could be obtained. The suspension was sonicated to allow particle disaggregation and prevent further agglomeration. The UV-vis absorption spectrum for this suspension was measured, and the spectral extinction coefficient was retrieved. The particle concentration in the suspension was then obtained from the spectral extinction coefficient using the Lambert-Beer law, known as the spectral absorption cross section of soot from the literature^41^,^42^,^43^. The mass concentration was determined assuming a soot density of 1.8 g/cm^3^. After 24 hours, we observed a deposit of soot particles and a decrease in the concentration of soot in UV-vis absorption measurements performed at intervals of 6 hours. To obtain a stable suspension, we decided to add 10% dimethyl sulfoxide (DMSO) to the suspension and, after sonication, we did not observe any deposits after 72 hours. UV-vis absorption confirmed that the concentration of soot remained constant after 72 hours as well. Dynamic light scattering was thus used to determine the size distribution of the particles effectively suspended in water/DMSO solution. The sizes of the suspended particles ranged from 20 nm up to approximately 300 nm, with mean sizes ranging from 80 to 120 nm.

### Cytokine measurements

Interleuchin (IL) -1α, IL-1β, IL-33, IL-18, IL-10, tumor necrosis factor (TNF) -α and IL-6 were measured in cell-free supernatants using commercially available enzyme-linked immunosorbent assay kits (ELISAs) (eBioscience, CA, USA).

### MTT assay

To assess cell viability, 3-(4,5-dimethylthiazol-2-yl)-2,5-diphenyltetrazolium bromide (MTT) was added to medium-free cells post treatment. DMSO was used to dissolve the purple formazan crystals. The formazan concentration was determined by measuring the optical density. The data are presented as absorbance (550 nm) vs treatment.

### Statistical Analysis

Data are reported as the mean ± standard error media (SEM). Statistical differences were assessed with one-way Analysis of variance (ANOVA) followed by Bonferroni’s multiple comparison post-test, and p values less than 0.05 were considered significant.

## Additional Information

**How to cite this article:** De Falco, G. *et al*. Human peripheral blood mononuclear cells (PBMCs) from smokers release higher levels of IL-1-like cytokines after exposure to combustion-generated ultrafine particles. *Sci. Rep.* **7**, 43016; doi: 10.1038/srep43016 (2017).

**Publisher's note:** Springer Nature remains neutral with regard to jurisdictional claims in published maps and institutional affiliations.

## Supplementary Material

Supplementary Figures
