# Supplementary material for: Human peripheral blood mononuclear cells (PBMCs) from smokers release higher levels of IL-1-like cytokines after exposure to combustion-generated ultrafine particles
Source: Sci Rep. 2017 Feb 22;7:43016. doi: 10.1038/srep43016 (PMC5320442; doi:10.1038/srep43016)
Supplement: Supplementary Figures [file srep43016-s1.doc]

**Human peripheral blood mononuclear cells (PBMCs) from smokers release higher levels of IL-1-like cytokines after exposure to combustion-generated ultrafine particles.**

Gianluigi De Falco^1,3^, Michela Terlizzi^2^, Mariano Sirignano^1^, Mario Commodo^3^, Andrea D’Anna^1^, Rita P. Aquino^2^, Aldo Pinto^2^, Rosalinda Sorrentino^2^

**Supplementary Figures**


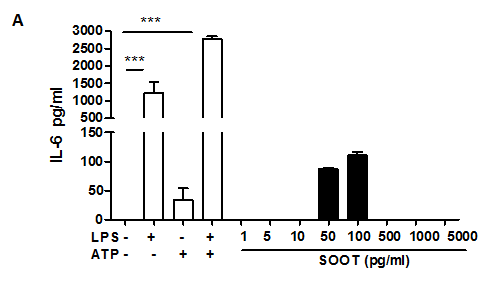


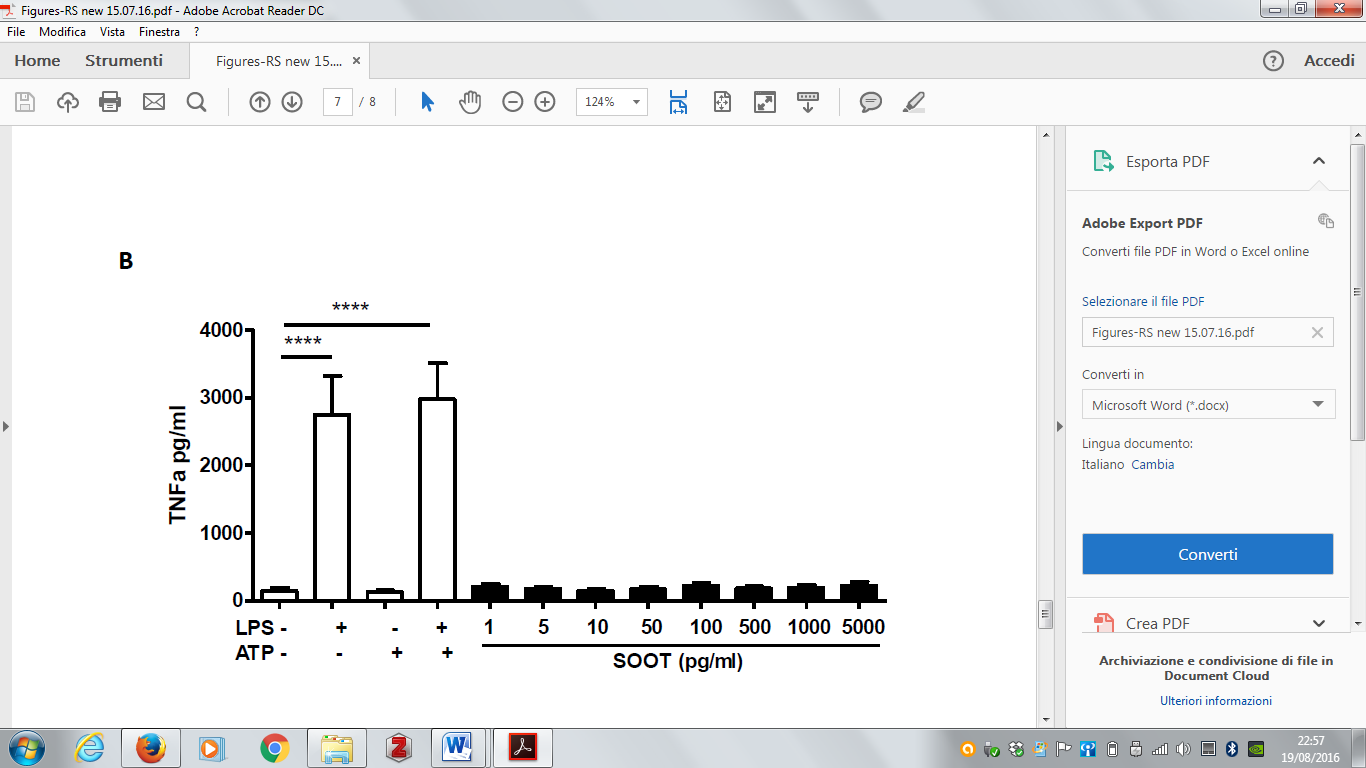


**Supplementary Figure 1. The administration of soot particles for 24 hours did not induce the release of IL-6 and TNF-α by murine macrophages.** J774.1 cells were treated with soot particles in a concentration dependent manner (1pg/ml-5ng/ml) for 24 hours. LPS (0.1µg/ml) and or ATP (0.5 mM) were used as positive control. The addition of soot particles onto macrophages did not induce the release of IL-6 (**A**) and TNF-α (**B**).

Data represent means ± SEM (n=12). Statistically significant differences are denoted by *** indicating p<0.001 as determined by One Way ANOVA followed by Bonferroni’s multiple comparison post test.


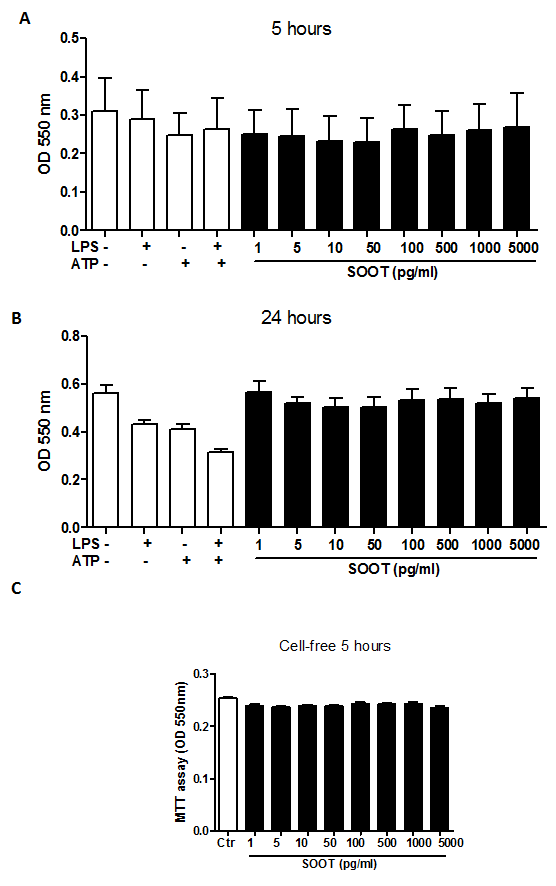


**Supplementary Figure 2. The administration of soot particles did not induce murine macrophages to cell death.** J774.1 cells were treated with soot particles in a concentration dependent manner (1pg/ml-5ng/ml) for 5 (**A**) and 24 hours (**B**). LPS (0.1µg/ml) and or ATP (0.5mM) were used as positive control. The addition of soot particles onto macrophages did not induce macrophages to cell death at both time points considered. Moreover, soot particles did not alter the absorbance of the MTT assay in a cell-free medium at 550 nm compared to the absorbance of the sole medium (C).

Data represent means ± SEM (n=12).
